# Generation-Common and -Specific Factors in Intention to Leave among Female Hospital Nurses: A Cross-Sectional Study Using a Large Japanese Sample

**DOI:** 10.3390/ijerph15081591

**Published:** 2018-07-26

**Authors:** Maki Tei-Tominaga, Kyoko Asakura, Takashi Asakura

**Affiliations:** 1 Faculty of Nursing, Setsunan University, Osaka 573-0101, Japan; 2 Division of Nursing Education and Administration, Graduate School of Medicine, Tohoku University, Sendai 980-0872, Japan; asakura@med.tohoku.ac.jp; 3 Laboratory of Health and Social Behaviour, Tokyo Gakugei University, Tokyo 184-8501, Japan; asakurat@u-gakugei.ac.jp

**Keywords:** female, generation, hospital, intention to leave, nurse

## Abstract

An understanding of the conditions that determine the factors affecting nurses’ intention to leave is important for countries suffering from nurse shortage. Aim: to examine factors influencing intention to leave among female hospital nurses in a large Japanese sample, classified into four generations by age and considering economic conditions. Methods: a cross-sectional survey with convenience sampling was conducted. Anonymous self-administered questionnaires were distributed to all nurses in 30 hospitals. To assess intention to leave, basic attributes, life conditions, work characteristics, and factors of psychosocial work environment were addressed. After classifying data into four generations based on age cohorts, we conducted multivariate logistic regression analysis using the completed data (*N* = 5074, mean age = 36.24 years). Results: regardless of the generational characteristics influenced by economic conditions, effort and monetary reward were generation-common factors. Over-commitment, social support, and the presence of a role model were generation-common factors in three generations. While having children increased intention to leave in the generation born in 1965–1979, having family members in need of caregiving other than children decreased the risk in the generation born in the 1980s. Conclusion: generational countermeasures considering factors of psychosocial work environment and life conditions are needed to avert female nurse turnover.

## 1. Introduction

The shortage of nurses is a concern in many countries [1]. LeVasseur et al. [2] stated that the registered nurse shortage has been a recurring concern for over five decades in the United States. In Japan, due to the labour-force shortage caused by an aging society, the shortage of nurses has become a critical issue. According to a 2017 national white paper [3], the proportion of the population above the age of 65 years in Japan was 27.3%, which is more than 10% higher than the average among the 35 countries of the Organisation for Economic Co-operation and Development (OECD) and more than two times higher than that of the Asia-Pacific region in general [4].

The Japanese government has estimated that about two million nursing professionals are needed to meet the demand, which will peak in 2025 when Japanese baby boomers (born in 1946–1949) will be at least 75 years old [5]. The number of employed nursing professionals increased by 0.3 million from 2005 to 2014 due to efforts by the Japanese government and relevant organisations [6]. Additionally, the turnover ratio of hospital nurses has remained stable (at 11%) since 2009 [7]. However, the Japanese government has noted that there is still a shortage of approximately 500,000 nurses [8].

In many countries, the healthcare industry is a female-dominated occupation [9], and Japan is no exception. In 2016, the medical and welfare industries were the most common industries for women in Japan, accounting for 6.07 million female workers out of the total female workforce of 57.45 million [10]. In addition, nurses are the most populous group among hospital employees, and, as of 2014, 94% of the nurses in Japan were women [6]. Therefore, to develop countermeasures to address the nursing shortage, further research is needed on the factors contributing to female nurses’ intention to leave, which is the strongest predictor of turnover [11].

Meanwhile, the age-specific employment rate of female employees in Japan is unique compared to other developed countries [12]. Traditionally, the line graph of the age-specific employment rate of Japanese females has shown an M-shaped curve, with the lowest ratio being among women in their thirties. This implies that women leave their jobs because of marriage and child-rearing; then, often, they resume full-time work after their children reach school age [12]. Regarding female nurses, however, the age-specific employment rate has not been an M-shaped curve, but one that has transformed over time. In 2002, the curve showed that the highest age-specific employment ratio was for those in their twenties (34%); however, in 2012, the highest age-specific employment ratio of female nurses was for those in their thirties (31%) and forties (26%) [13].

This trend among female nurses in Japan might derive not only from the abovementioned female-specific life events (marriage, child-rearing, etc.) but also from the ‘generational cohort’. This term refers to a group with similar birth years, history, and life experiences. They also tend to have similar attitudes, emotions, belief, values, and preferences regarding work and career [14]. Research on nurses’ turnover rates requires the consideration of generational characteristics, since the misunderstanding of differences inherent in a multigenerational workforce (e.g., generational values, work ethic) can contribute to conflicts in the nursing workplace [15], which influence nurse retention. Hayes et al. also noted that the factors that influence nurses’ decisions to leave their jobs differ according to generation [16].

Economic conditions, in particular, can influence values and behaviours in relation to work. Brewer et al. [17] found that the age cohort of new registered nurses who were licensed after the recession showed higher commitment to their employers than those who were licensed before the recession, although neither nurses’ incomes nor their reported job satisfaction levels had changed. Psychosocial work environment can affect turnover and nurses’ intention to leave. These include effort–reward imbalance [18], work climate [19], social support [20,21,22], the presence of a role model [21,23], and job control [22]. However, these factors may differ by generation according to whether they have experienced or remember certain, especially disruptive historical or socioeconomic events, such as the recession following the collapse of Japan’s bubble economy [24].

Moreover, life conditions related to female-specific life events and work characteristics (e.g., working hours, job position) may also differently influence female nurse turnover according to generation. In Japan, having and raising children is the most-listed reason for resignation among all female workers in their twenties and thirties [12], including nurses [8]. Additionally, caregiving for family members other than children was given as a reason for resignation among female employees over fifty; this is because females account for about 70% of primary caregivers in Japanese families with members in need of care [12]. On the other hand, 80% of poll respondents endorsed women’s continued employment, which has remained high in the past few years in Japan [25].

In Western countries, several conventional generations have been specified in the workforce: baby boomers, born between 1946 and 1964; generation X, born between 1965 and 1979; and generation Y, born between 1980 and 1995 [26]. A previous study [27] suggested that generational work values differ and that work values change as people grow older, with an increasing desire among American workers to balance work and life. Such generational differences in work attitude (e.g., intent to continue working, job satisfaction, intention to quit) are also observed among hospital workers and nurses [19,28,29].

In Japan, one study [30] examined the factors affecting nurses’ intention to leave using combined-sex data classified by three generations per specific definitions (e.g., the generation born between 1947 and 1949 is defined as Japanese baby boomers, etc.). The abovementioned characteristics of baby boomers in Western countries are also observed in the generation born in Japan between 1950 and 1969 [31], which reflected societal conditions. However, the findings could not be generalised to other nursing populations because of the small sample size (*N* = 315) and low return rate (39%). Additionally, the generational classifications in that study did not consider economic background, which influences the work values of each generation [24].

On the other hand, the factors influencing intention to leave could be similar between nurses in Japan and those in Western countries, since each generation may share common environments, such as the global economy. Consequently, this study aimed to examine generation-common and generation-specific factors (e.g., factors of psychosocial work environment, life conditions related to female-specific life events, work characteristics) affecting intention to leave among Japanese female hospital nurses, classifying them by age cohort and considering domestic economic conditions.

## 2. Materials and Methods

### 2.1. Design, Participants, and Settings

We conducted quantitative research using a cross-sectional survey with convenience sampling. To control potential confounding factors (organisational culture, benefit package, etc.), we chose hospitals from a group institution that has 94 hospitals throughout Japan. The institution has more than 130 years of history providing medical aid activities and is characterised by similar organisational policies and cultures (e.g., humanitarian mission, emergency relief). After receiving an explanation of the study from the researchers, the directors of the nursing departments from 30 hospitals located in western Japan, from which we requested cooperation, agreed and returned signed consent forms. All hospitals were ‘advanced treatment hospitals’, each comprising 100–999 beds and a nurse-to-patient ratio of 1:7. The turnover ratio of nurses in hospitals of this institution was 8.2% in both 2012 and 2013, which was relatively lower than the national average (11%) [7].

### 2.2. Data Collection

In January 2014, anonymous self-administered questionnaires were distributed to all nurses in the 30 hospitals (*N* = 11,171). If participants agreed to cooperate, they completed and returned their questionnaire to the researchers in a sealed envelope via the nursing department within one to four weeks of distribution. For participants who wished to answer via a web-based questionnaire, we concurrently provided the survey online, while maintaining confidentiality of personal information. No reminder was sent to participants after the first notice of the study.

More than half (*N* = 5763, 51.6%) of the 28 hospitals returned their questionnaires. There were no responses from the other two hospitals, which did not distribute questionnaires to nurses due to the issue of management for cooperation of this study. After excluding incomplete responses for age, sex, and current job license, we also excluded data from men (5%), those born before 1949 (over 65 years old), public health nurses, midwives, and assistant nurses. Then, we classified the remaining nurses’ data (*N* = 5106) into four generations by age cohort, considering domestic economic conditions [24]. Comparing our sample with the representative hospital nurse population of Japan in 2014 (percentage of male hospital nurses = 6.8%; age groups were 20.6% for 50–64 years old, 42.8% for 35–49 years old, 26.7% for 25–34 years old, and 8.2% for <24 years old) [6], we confirmed that our sample had more female nurses and younger generation groups. For the final statistical analyses, we used the completed data (*N* = 5074) after excluding missing data for basic attributes and the independent and dependent variables.

### 2.3. Definitions

We defined four generations based on year of birth, referring to an analysis report by the Japanese government [24] (See Appendix A). Those in the generation born between 1950 and 1964 experienced the heyday of the bubble economy as adults, those born between 1965 and 1979 remember the bubble economy from their adolescence/early adulthood, those born in the 1980s do not have memories of the bubble economy and experienced recession periods in adolescence, and those from the generation born after 1990 have memories of economic fluctuation from adolescence.

Researchers in Western countries have noted the characteristics of different generations [19,27,29]: baby boomers (strong values, loyalty, work ethic, recognition from authority, etc.), generation X (arguably the best-educated generation, with most achieving higher educational attainment than their parents; loyal to their jobs without compromising work–life balance; etc.), and generation Y (had consistent access to information–communication technology in their youth; prefer immediate rewards; thrive in working environments that offer flexibility, recognition, and respect; etc.).

In Japan, meanwhile, the generation born between 1947 and 1949 is defined as Dankai Sedai (Japanese baby boomers). The abovementioned characteristics of baby boomers in Western countries are also observed in the generation born in Japan between 1950 and 1969 [31]. Additionally, many characteristics of generation Y are observed in the generation born after 1990, and in Japanese society they are known for certain characteristics, such as a need for approval and a lack of aspiration. They are called Yutori Sedai (relaxed generation), since they grew up in a sophisticated information technology environment and experienced a more relaxed educational curriculum—‘Yutori education’ [32].

### 2.4. Measures

The questionnaire comprised items on life conditions related to female-specific life events (i.e., having children, having family members in need of caregiving other than children) and work characteristics (employment status, working tenure, hours of overtime per week, frequency of work on days off, and job position)—which are considered issues that influence work–life balance and may cause nurses to leave their jobs [8,12]—and scales addressing factors of the psychosocial work environment (i.e., Japanese short version of the Effort–Reward Imbalance Questionnaire, Presence of a Role Model Scale, and Social Support in the Workplace Scale) were used as independent variables. We set intention to leave as a dependent variable and participants’ basic attributes (i.e., sex, age, marital status, educational level, employment status, job position) as control variables.

#### 2.4.1. Factors of the Psychosocial Work Environment

We employed the Japanese short version of the Effort–Reward Imbalance Questionnaire (ERI), developed by Siegrist and colleagues, to assess stressful experiences in the work environment [33,34]. The ERI, which has high internal reliability and validity for a range of working populations [33], includes an examination of female hospital nurses [18]. The short version of the ERI comprises two scales that measure extrinsic components: effort (six Likert-type items), which refers to the demanding conditions employees face in the work environment, and reward (11 Likert-type items with three subscales: money reward, esteem reward, and career opportunity reward), which refers to the three transmitters of reward for employees. The items that measure extrinsic components were quantified in two steps using Siegrist’s ERI and scoring method. After confirming the data through preliminary analysis, we did not use the effort–reward ratio, which did not show significance; rather, we used each variate value as an independent variable to examine the effect on the dependent variable in the final analyses. Regarding effort, higher total scores indicate greater distress over workload/effort. Regarding reward, higher total scores indicate greater reward received by the participant.

The ERI also includes a scale addressing over-commitment (six Likert-type items), which is an intrinsic personal dimension capturing ways of coping with demanding situations and of eliciting extrinsic rewards. Higher values indicate that the participant is easily overwhelmed by time pressure at work or that she has problems relaxing and disconnecting from work when not at work.

#### 2.4.2. Presence of a Role Model

The presence of a role model at work has previously been shown to have a significant relationship with intention to leave among newly graduated nurses in Japan [22,23,35]. To assess the presence and influence of role models in relation to intention to leave, we used one item (‘There is a nurse I work with who represents a professional ideal for me’) from the Role Model Scale [35], which was evaluated on a four-point Likert-type scale. A higher score indicated the greater presence and influence of role models.

#### 2.4.3. Social Support in the Workplace

Social support in nurses’ workplaces was assessed using a three-item original scale, which was developed by the researchers after referring to previous studies [21,22,36]. Each item asked for the participants’ perceptions of supportive conditions at work (e.g., ‘In our workplace, members help each other in their work’). All items were evaluated using a four-point Likert-type scale, with higher scores indicating greater social support at work.

#### 2.4.4. Intention to Leave

Intention to leave was assessed using a six-item scale that had showed high internal consistency, reliability, and validity for information technology service employees and nurses in Japan [22,23,35,37]. Each item asked about participants’ thoughts or behaviours related to resigning from their jobs. Responses were scored on a four-point Likert-type scale, with higher scores representing greater intention to leave. As employees’ intentions to leave are the greatest predictor of turnover [11], nurses with a score in the upper quartile of intention to leave (≥18) were defined as a group at considerable risk to leave referring to the previous study [21].

### 2.5. Ethical Considerations

Approval for this study was obtained from the institutional ethics committee at the second author’s institution (#2013-1-144) in 2013. Participants were informed about the voluntary nature of participation and assured of confidentiality in the handling of data.

### 2.6. Analysis

We calculated descriptive statistics for the basic attributes, life conditions, and work characteristics of the sample. Next, we calculated the descriptive statistics and Cronbach’s alphas of the independent and dependent variables, as well as the correlation coefficients between the dependent variable (i.e., the upper quartile of intention to leave score) and each variable, as a preliminary analysis. Then, we performed multivariate logistic regression analysis for the dependent variable in each generation. All factors of the psychosocial work environment were entered into an equation along with life conditions related to female-specific life events and work characteristics, controlling for basic attributes. The odds ratio (OR) and 95% confidence interval (CI) of each control and independent variable were calculated for the dependent variable. A *p*-value of less than 0.05 was regarded as indicating statistical significance.

## 3. Results

### 3.1. Participants Characteristics

Participants were classified into four generations (Table 1): 13% were of the generation born between 1950 and 1964 (≥50 years old at the time of data collection), 38% were of the generation born between 1965 and 1979 (35–49 years old), 35% were of the generation born in the 1980s (25–34 years old), and 14% were of the generation born after 1990 (<24 years old). Among the four generations, those born between 1950 and 1964 showed the highest ratio of nurses who worked on scheduled days off per month (40%) and the highest ratio of nurses who worked 10 or more hours per week of overtime (22%).

### 3.2. Descriptive Statistics and Reliability of Each Scale and the Association between Dependent Variables

Mean scores for intention to leave in decreasing order were for the generation born during the 1980s (14.53 ± 4.83), after 1990 (13.65 ± 5.00), between 1965 and 1979 (13.47 ± 4.80), and between 1950 and 1964 (12.88 ± 4.53) (Table 2). Although not shown in Table 2, mean scores for intention to leave of the generation born between 1950 and 1964 were significantly lower than the mean scores of those born between 1965 and 1979 (*p* < 0.05), those born during the 1980s (*p* < 0.001), and those born after 1990 (*p* < 0.05).

Regarding alpha coefficients, intention to leave was 0.89–0.90; the subscales of the ERI were 0.89–0.92 for effort, 0.79–0.81 for money reward, 0.88–0.90 for esteem reward, 0.58–0.64 for career opportunity reward, 0.56–0.61 for over-commitment, and 0.85–0.91 for social support.

### 3.3. Results of Correlation Coefficients between the Dependent Variable and Each Variable

As shown in Table 3, correlation coefficients between the dependent variable (upper quartile of the intention to leave score) and each variable of the psychosocial work environment were shown to have significant associations (*p* < 0.001) for all generations, whereas effort and over-commitment showed weak to moderate negative correlated coefficients, and money reward, esteem reward, career opportunities reward, social support, and role model showed weak to moderate positively correlated coefficients. On the other hand, the correlated coefficients between the dependent variable and variables of life conditions related to female-specific life events (i.e., having children and having family members in need of nursing care) showed weak significance in two generations (i.e., those born between 1965 and 1979 and those born in the 1980s).

### 3.4. Results of the Multivariate Logistic Regression Analysis

As generation-common risk factors of intention to leave, effort (odds ratio = 1.06–1.13) in all generations and over-commitment (OR = 1.12–1.25) in three generations (those born in 1965–1979, during the 1980s, and after 1990) showed significance. Regarding generation-common factors reducing intentions to leave, money reward (OR = 0.85–0.92) in all generations, social support (OR = 0.80–0.85) in three generations (those born in 1965–1979, during the 1980s, and after 1990), and the presence of a role model (OR = 0.69–0.83) in three other generations (those born in 1950–1964, 1965–1979, and during the 1980s) showed significance.

As generation-specific risk factors of intention to leave, overtime of more than 15 h per week (OR = 2.02) and having children (OR = 1.59) showed significance in the generation born in 1965–1979. Regarding generation-specific factors reducing intentions to leave, career opportunity reward (OR = 0.68) showed significance for the generation born in 1965–1979, while having family members in need of caregiving other than children (OR = 0.58) showed significance for the generation born during the 1980s (Table 4).

## 4. Discussion

This study identified generation-common and generation-specific factors influencing intention to leave among female hospital nurses. Regardless of the characteristics of generations influenced by different domestic economic conditions, effort and over-commitment were generation-common risk factors in intention to leave, and factors reducing intentions to leave among female hospital nurses were the importance of money reward, the presence of a role model at work, and social support. Findings also revealed generation-specific factors in intention to leave among female hospital nurses.

### 4.1. Generation-Common Factors

The mean score for intention to leave was lowest in the generation born between 1950 and 1964, and it was significantly lower than that of any of the other three generations. However, this generation also had the highest ratio of nurses who worked on scheduled days off per month and the highest ratio of nurses who worked 10 or more hours of overtime per week. This finding may derive from the characteristics and work values of the generation (having strong values, loyalty, a strong work ethic, seeking recognition from authority, etc.), which are shared by both Western countries and Japan [19,25,31]. A study of generational differences that compared 27–40-year-olds in 1974 to 27–40-year-olds in 1999, and compared 41–65-year-olds in the same time frame, found that employees had become less convinced that work should be an important part of life; this change in values was reflected even within cohort groups as they aged [27]. Additionally, nursing studies in Western countries noted generational differences in attitudes and characteristics [19,28,29]; for example, generation Y obtained significantly lower scores on the ‘Challenge’ scale than baby boomers [19]. The Japanese government also reported similar conditions [24].

Our findings add new insights regarding the risk factors in intention to leave among female hospital nurses in Japan. For example, both effort and over-commitment were important factors, as in Western countries. The findings related to effort are similar to a previous study that found that workload-related disincentives that encourage nurses to leave hospital employment were selected most frequently across all three generational cohorts (*N* = 9904) [38]. Additionally, the finding that over-commitment elevated intention to leave in three generations (those born between 1965 and 1979, in the 1980s, and after 1990) might be specific to female hospital nurses. Previous studies have found that, among healthcare personnel (nurses and physicians), women scored higher on work-related over-commitment compared to men [39]. Thus, our findings on this issue of over-commitment in three generations of female hospital nurses require additional research attention.

In terms of factors reducing intentions to leave, our findings revealed the consistent significance of money reward regardless of generational characteristics influenced by different domestic economic conditions. The findings related to money reward are consistent with earlier research addressing seven European countries [18], which found an elevated intention to leave among female hospital nurses who had ‘reward frustration’, which had the strongest explanatory power. However, the findings regarding money reward could have been influenced by the conditions of Japanese medical institutions. In Japan, the line graph of nurses’ age-specific hourly wages reveals a gentler curve than that of university-educated female employees [7,12], meaning there is less of a wage increase. Additionally, their average monthly salaries (¥270,506, roughly equivalent to US $2427, for a newly university-graduated nurse and ¥318,117, or US $2865, for a staff nurse who has worked 10 years; 22 June 2018 exchange rates) had not increased in the five years since 2011 [7]. This implies that remuneration revisions for medical services in Japan were not reflected in the salaries of nursing staff.

On the other hand, pecuniary consideration, such as money reward, is not always a feasible option for most hospitals, depending on their financial position. The present study revealed two factors which have decreased risk of intentions to leave: social support, which was a common generational factor in three generations (those born between 1965 and 1979, in the 1980s, and after 1990), and the presence of a role model, which was a common generational factor for all generations except the one born after 1990. These findings support previous findings regarding social support [20,21,22] and the presence of a role model [21,23], and highlight the need to strengthen human resource support and social support in the workplace, which could be acceptable countermeasures at the hospital level for reducing intention to leave among female nurses.

### 4.2. Generation-Specific Factors

Regarding the generation-specific risk factors in intention to leave, in the present study, findings on life conditions related to female-specific life events were different according to generation. Having children (OR = 1.59, CI: 1.13–2.25) showed a significant relationship with intention to leave only in the generation born between 1965 and 1979 (35–49 years old).This finding supports a Japanese government survey [8] that reported that bearing and raising children (22.1%) was the main reason for nurses quitting their first job.

In terms of factors reducing intentions to leave, career opportunity reward (OR = 0.68, CI: 0.56–0.83) also showed a significant relationship with intention to leave only in the generation born between 1965 and 1979 (35–49 years old). Our findings suggested that, compared to baby boomers, generation X reported more negative experiences [40] and experienced their work settings as less consistent with their personal values [41].

In Japan, caregiving and nursing was a commonly listed reason for resignation among female employees. Women comprise about 70% of the primary caregivers in families that have members in need of nursing care. Additionally, 13.5% of employed female nurses take on the role of caretaker for a family member in need, whereas 45% of unemployed female nurses take on this role [12]. Considering the findings that generation X and Y nurses desire flexible working arrangements to achieve an acceptable work–life balance [28], and nurses providing elder care at home who would be more fatigued and sleep-deprived [42], it would be possible that having family members in need of caregiving other than children is a risk factor in intention to leave. However, in the present study, it was a factor reducing intention to leave (OR = 0.58, CI: 0.40–0.84) in the generation born in the 1980s (25–34 years old). Our contradictory findings might have been influenced by the successful management of their dual roles both as staff nurses and individuals who provide care for family members in need other than children (e.g., identifying units and shifts that suit their needs, creating clear boundaries between home and work) [43]. Also, a supportive work environment for the participants in this study might have allowed them to continue working, even under difficult family circumstances.

The findings also highlight the unique characteristics of the generation born after 1990 (<24 years old at the time of data collection). The findings on the presence of a role model in this study contrasts with previous findings that the presence of a role model consistently influences intention to leave and decision to resign among newly graduated Japanese nurses who were hired in 2008–2009 (categorised in this study within the generation born during the 1980s) [21,23]. This might be influenced by the unique characteristics of ‘Generation Yutori’ (sophisticated media and computer environment, lacking ambition, etc.) [32,44]. According to a 2013 Japanese white paper [25], the number of Internet users in Japan expanded by 8.3 times in the 13 years after 1997, social media use has become widespread, and especially among young people, communication behaviour has diversified. A previous study [45] also revealed the behavioural influence of social media, particularly Facebook. The influence of Facebook is that the platform provides quick connection: people can reach anyone, bonding across cultures and distances through the network, presenting the best side of themselves, and showing off their accomplishments to everyone they friended on Facebook. Our findings could have been influenced by this rapid spread of the Internet and social media, which has enabled this generation to easily befriend people who could become role models. Thus, lacking a role model in the workplace might matter less for this generation than for older generations.

In many countries, including Japan, the healthcare industry is a female-dominated occupation [9,10]. Moreover, the shortage of nurses is a concern in many countries [1,2]. Previous studies in Western countries have suggested that generational work values and attitudes differ and change as people grow older [27]. Such generational differences in work attitude (e.g., intent to continue working, job satisfaction, intention to quit) were also observed on not only in Western countries’ hospital workers and nurses [19,28,29] but also in our participants, Japanese female hospital nurses. The age composition of staff nurses varies with each hospital; thus, our findings on the generation-common risk factors (i.e., effort and over-commitment) and the factors reducing intentions to leave (i.e., money reward, the presence of a role model, and social support) among female hospital nurses will provide fruitful suggestions for managers and employers in hospital workplaces with different age compositions when considering countermeasures to avert nurse turnover.

This study had some limitations. First, the participants were female nurses in hospitals from a group institution that shared similar organisational policies and cultures in Japan, making the results possibly unique to the participants in this study. Second, the response rate was relatively modest, and some scales showed relatively low reliability. The validity of the original three-item scale for social support in nurses’ workplaces should also be examined. Third, this study used a cross-sectional design; therefore, longitudinal surveys are needed to determine the causal relationships between actual turnover and work environment/life condition factors. Since hospital nursing is a female-dominated occupation in many countries, understanding the generation-common and -specific factors of the psychosocial work environment and life conditions related to female-specific life events is needed to counter nursing shortages. However, despite these limitations, our findings on female hospital nurses across four generations provide meaningful new insights for addressing the critical issue of nurse shortage in medical institutions in Japan’s aging society.

## 5. Conclusions

Using a large Japanese sample classified into four generations, this study examined the generation-common and generation-specific factors in intention to leave among female hospital nurses. Regardless of the generational characteristics influenced by different domestic economic conditions, effort and money reward, both which are factors of the psychosocial work environment, were generation-common factors. Over-commitment, social support, and the presence of a role model were influencing factors for intention to leave for three of the four generations. Regarding life conditions, while having children increased intention to leave in the generation born in 1965–1979, having family members in need of caregiving other than children reduced intention to leave in the generation born during the 1980s. Generational countermeasures that consider nurses’ psychosocial work environment and life conditions related to female-specific life events are needed to avert female nurse turnover and a future nursing labour-force shortage in this aging society.

## Figures and Tables

**Table 1:** Basic attributes of participating female hospital nurses (*N* = 5108) ^a^.

Items	Category	Born in 1950–1964 (over 50 Years Old)		Born in 1965–1979 (35–49 Years Old)		Born during the 1980s (25–34 Years Old)		Born after 1990 (Less Than 24 Years Old)	
		*N*	%	*N*	%	*N*	%	*N*	%
1. Marital status	Single	127	19	643	34	1148	64	673	97
	Married	556	81	1269	66	638	36	20	3
2. Education	Junior college or vocational school	625	92	1735	91	1215	68	446	64
	University	6	1	38	2	464	26	232	34
	Graduate school	13	2	27	1	15	1	2	0
	Others	39	6	112	6	92	5	13	2
3. Employment status	Non-regular employee	70	10	180	10	57	3	2	0
	Regular employee	613	90	1730	90	1727	97	691	100
4. Having children	Yes	559	82	1216	64	1375	77	1	0
	No	124	18	696	36	411	23	692	100
5. Having family members in need of caregiving other than children	Yes	223	33	321	17	176	10	50	7
	No	458	67	1591	83	1610	90	643	93
6. Job position	Staff nurses/senior staff nurses	398	58	1521	80	1786	100	693	100
	Status equal to head nurses	161	24	109	6	0	0	0	0
	Nursing directors or vice-directors	124	18	282	15	0	0	0	0
7. Frequency of working on scheduled days off per month	None	411	60	1263	67	1171	66	465	67
	1–2 days	184	27	438	23	412	23	159	23
	3–4 days	57	8	140	7	143	8	50	7
	5–6 days	21	3	55	3	45	3	17	3
	7 days or more	10	2	16	1	15	1	2	0
8. Average overtime work hours per week	None	96	14	186	10	129	7	37	5
	1–4 h	292	43	937	49	909	51	287	41
	5–9 h	143	21	483	25	482	27	213	31
	10–14 h	90	13	159	8	181	10	103	15
	15–19 h	30	4	68	4	40	2	29	4
	20 h or more	32	5	79	4	45	3	24	4
	Mean (SD)								
9. Age (years)		54.23 (3.13)		41.38 (4.24)		29.91 (7.10)		22.99 (0.86)	
10. Job tenure		31.47 (4.88)		18.23 (5.55)		2.87 (3.07)		1.83 (0.90)	

^a^ Including missing data.

**Table 2:** Descriptive statistics for dependent variable and independent variables.

Variables	Items	Sample Range	Mean (SD)			
			Born in 1950–1964	Born in 1965–1979	Born during the 1980s	Born after 1990
			(*N* = 673)	(*N* = 1912)	(*N* = 1786)	(*N* = 693)
Intention to leave	6.00	6–24	12.88 (4.53)	13.47 (4.80)	14.53 (4.83)	13.65 (5.00)
Psychosocial work environment						
Effort ^a^	6.00	6–30	17.47 (5.50)	18.44 (5.43)	18.58 (5.09)	18.35 (5.15)
Money reward ^a^	4.00	4–20	14.11 (3.34)	13.83 (3.34)	14.27 (3.34)	15.0 (3.31)
Esteem reward ^a^	5.00	7–25	14.39 (3.51)	14.63 (3.55)	15.31 (3.48)	15.66 (3.33)
Career opportunity reward ^a^	2.00	2–8	2.81 (0.74)	2.82 (0.71)	2.76 (0.72)	2.94 (0.70)
Over-commitment ^a^	5.00	6–24	14.29 (2.67)	14.17 (2.75)	14.12 (2.67)	14.49 (2.70)
Social support ^b^	3.00	3–12	9.05 (1.74)	8.99 (1.77)	8.99 (1.79)	9.23 (1.74)
Role model ^c^	1.00	1–4	2.55 (0.77)	2.61 (0.78)	2.66 (0.78)	2.95 (0.75)

^a^ The short version of the Effort–Reward Imbalance Questionnaire (ERI). Scale questionnaire consists of effort, reward (three subscales: money reward, esteem reward, and career opportunity reward), and over-commitment. A higher score indicates a greater degree of the characteristic in the participant. ^b^ A higher total score indicates greater social support at work. ^c^ A higher score indicates the greater presence and influence of role models.

**Table 3:** Spearman’s correlation coefficients between the dependent variable and each variable.

	The Upper Quartile of Intentions to Leave Score ^a,b^							
	Born in 1950–1964		Born in 1965–1979		Born during the 1980s		Born after 1990	
**Basic attribute**								
Age	−0.100	**	−0.076	***	−0.021		0.096	*
Marital status ^c^	−0.044		−0.086	***	−0.089	***	−0.014	
Education ^d^	−0.005		−0.013		0.007		−0.065	
Regular employee ^e^	−0.040		0.014		−0.039		0.025	
Managerial job position ^e^	−0.074		−0.067	**	0.013		−	
**Life conditions related to female-specific life events**								
Having children ^e^	−0.020		−0.118	***	−0.100	***	0.002	
Having family members in need of nursing care ^e^	0.025		0.055	*	0.108	***	0.029	
**Work characteristics**								
No work on day off ^e^	−0.030		−0.001		−0.036		−0.093	*
Overtime less than 4 h per week ^e^	0.017		−0.051	*	−0.104	***	−0.100	*
Overtime more than 15 h per week ^e^	0.020		−0.036		0.060	*	0.128	**
**Psychosocial work environment**								
Effort ^f^	0.205	***	0.291	***	0.355	***	0.479	***
Money reward ^f^	−0.274	***	−0.312	***	−0.370	***	−0.460	***
Esteem reward ^f^	−0.214	***	−0.278	***	−0.330	***	−0.417	***
Career opportunities reward ^f^	−0.183	***	−0.215	***	−0.166	***	−0.331	***
Over-commitment ^f^	0.213	***	0.257	***	0.315	***	0.446	***
Social support ^f^	−0.176	***	−0.238	***	−0.272	***	−0.311	***
Role model ^f^	−0.181	***	−0.207	***	−0.181	***	−0.244	***

^a^ 1 = the upper quartile of intentions to leave score, others = 0. Symbols indicate level of significance: ^b^ *p* < 0.10, * *p* < 0.05, ** *p* < 0.01, *** *p* < 0.001. ^c^ 1 = Married, 0 = Single. ^d^ 1 = College graduate or higher, 0 = Junior college or vocational school equivalency degree. ^e^ 1 = agree, 0 = disagree. ^f^ Continuous variable.

**Table 4:** Results of multivariate logistic regression for the upper quartile of intention to leave scores among female participants in four generations ^a^.

Variables	Born in 1950–1964			Born in 1965–1979			Born during the 1980s			Born after 1990		
	Odds Ratio	95% Cl ^b^	*p* ^b^	Odds Ratio	95% Cl ^b^	*p* ^b^	Odds Ratio	95% Cl ^b^	*p* ^b^	Odds Ratio	95% Cl ^b^	*p* ^b^
**Life conditions related to female-specific life events**												
Having children ^c^	0.80	0.33–1.90		1.59	1.13–2.25	**	1.12	0.76–1.65		-	-	-
Having family members in need of caregiving other than children ^c^	1.13	0.71–1.80		0.75	0.55–1.03		0.58	0.40–0.84	**	1.23	0.57–2.62	
**Work characteristics**												
No work on day off ^c^	0.92	0.58–1.46		0.81	0.63–1.06		0.82	0.64–1.06		1.20	0.79–1.83	
Overtime less than 4 h per week ^c^	0.68	0.40–1.14		0.92	0.70–1.20		0.99	0.77–1.27		0.67	0.43–1.05	
Overtime more than 15 h per week ^c^	0.83	0.37–1.84		2.02	1.20–3.42	**	1.18	0.69–2.02		0.59	0.28–1.26	
**Psychosocial work environment**												
Effort ^d^	1.06	1.00–1.13	*	1.09	1.05–1.12	***	1.09	1.06–1.13	***	1.13	1.06–1.19	***
Money reward ^d^	0.85	0.77–0.94	**	0.92	0.87–0.98	**	0.88	0.84–0.93	***	0.87	0.80–0.96	***
Esteem reward ^d^	1.04	0.95–1.14		0.99	0.94–1.04		0.97	0.92–1.02		1.01	0.92–1.10	
Career opportunity reward ^d^	0.72	0.51–1.02		0.68	0.56–0.83	***	0.87	0.72–1.06		0.78	0.55–1.11	
Over-commitment ^d^	1.10	0.99–1.22		1.12	1.06–1.18	***	1.14	1.08–1.20	***	1.25	1.13–1.39	***
Social support ^d^	0.69	0.49–0.97	*	0.82	0.68–0.98	*	0.83	0.69–0.99	*	0.81	0.59–1.12	
Presence of a role model ^d^	0.95	0.81–1.10		0.84	0.78–0.91	***	0.85	0.78–0.92	***	0.80	0.69–0.93	***

^a^ The results of the multivariate logistic regression analysis for the upper quartile of the intention to leave score, adjusted for the age, marital status, and job position of each generation. Hosmer–Lomeshow goodness of fit: those born in 1950–1964, χ^2^ = 11.084, degree of freedom = 8, *p* = 0.197; born in 1965–1979, χ^2^ = 4.563, degree of freedom = 8, *p* = 0.803; born during the 1980s, χ^2^ = 10.925, degree of freedom = 8, *p* = 0.206; born after 1990, χ^2^ = 0.574, degree of freedom = 8, *p* = 0.676. ^b^ Cl; confidence interval, * *p* < 0.05, ** *p* < 0.01, *** *p* < 0.001. ^c^ 1 = agree, 0 = disagree. ^d^ Continuous variable.

**Figure A1:**
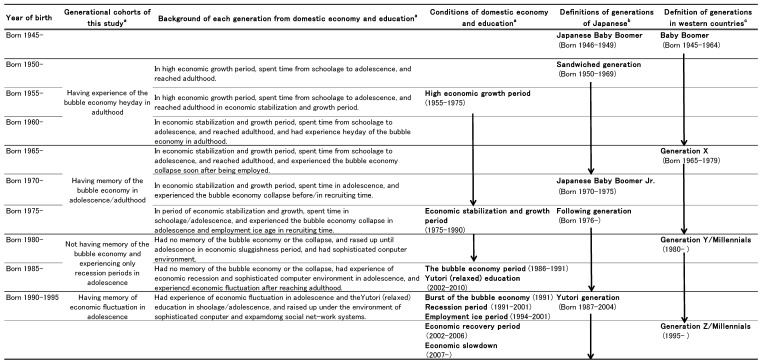
Four generational cohorts based on year of birth. ^a^ References [24], ^b^ References [30,31], ^c^ References [26,27].
